# Development of an eco‐friendly RNAi yeast attractive targeted sugar bait that silences the *shaker* gene in spotted‐wing drosophila, *Drosophila suzukii*

**DOI:** 10.1002/ps.70228

**Published:** 2025-09-18

**Authors:** Keshava Mysore, Jackson Graham, Teresia M. Njoroge, Akilah T. M. Stewart, Saisuhas Nelaturi, Molly Duman‐Scheel

**Affiliations:** ^1^ Department of Medical and Molecular Genetics Indiana University School of Medicine, Raclin‐Carmichael Hall South Bend Indiana USA; ^2^ Eck Institute for Global Health University of Notre Dame Notre Dame Indiana USA; ^3^ Department of Biological Sciences University of Notre Dame Notre Dame Indiana USA; ^4^ Department of Chemistry University of Notre Dame Notre Dame Indiana USA

**Keywords:** biopesticide, crop, eco‐friendly, fruit, insecticide, integrated pest management, SWD, targeted biopesticide

## Abstract

**Background:**

*Drosophila suzukii,* or spotted‐wing drosophila (SWD), (Diptera: Drosophilidae), are invasive vinegar flies of East Asian origin that have wreaked havoc on the small fruit and berry industry. In locations where SWD are well established, weekly chemical insecticide applications are necessary, resulting in increased economic costs, unwanted environmental impacts ensuing from loss of non‐targeted organisms, and the eventual emergence of populations that are resistant to these insecticides. It is therefore critical that new classes of biorational pesticides and cost‐effective technologies for controlling SWD are identified.

**Results:**

Here, we used the attractive properties of *Saccharomyces cerevisiae*, baker's yeast, which was designed to express an RNA interference (RNAi) pesticide that specifically targets the SWD *Shaker (Sh)* gene, to lure and kill flies that feed on the yeast, which was delivered in a feeder as a component of an attractive targeted sugar bait (ATSB). The yeast, which was heat killed prior to preparation of the ATSB, silenced the *Sh* gene, resulting in severe neural defects and 96 ± 9% fly mortality in laboratory trials. The RNAi yeast was successfully fed to the flies in an easily assembled soda bottle feeder that continuously rewetted the yeast with soda, which lured and killed the flies in simulated field trials. Despite this toxicity observed in SWD, consumption of the yeast had no impact on the survival of other dipteran insects.

**Conclusion:**

This promising ATSB technology, which was prepared with a new class of RNAi yeast insecticides, could one day be an effective component in integrated SWD control programs. © 2025 The Author(s). *Pest Management Science* published by John Wiley & Sons Ltd on behalf of Society of Chemical Industry.

## Graphical abstract

This study discusses a promising new attractive targeted sugar bait (ATSB) technology using yeast that expresses shRNA targeting essential genes in the fruit pest spotted‐wing drosophila (SWD). The yeast, which is delivered using soda as a sugar bait, could one day be an effective component in integrated SWD control programs.

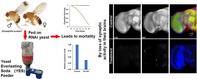

## 1 INTRODUCTION

*Drosophila suzukii*, or SWD, are invasive vinegar flies of East Asian origin. SWD, which lay eggs in a variety of wild and cultivated plants, have decimated the small fruit industry worldwide, including Europe, the Americas, and Africa.^1^, ^2^, ^3^ SWD, which complete multiple generations in a year, impact most berry crops, cherries, grapes and other tree fruits, generating upward of 80% crop loss, and an estimated $700 million economic loss for producers annually when they are not adequately controlled.^4^ SWD females penetrate the skin of fresh fruit prior to harvest, laying eggs under the skin, and compromising the fruit integrity, resulting in dimpling, wrinkling, and browning of the larval‐infested fruit as well as potential sites for infestation by other insects and infection sites for microbial pathogens.^2^ In regions where SWD are well established, weekly insecticide applications are necessary,^5^, ^6^ generating increased economic costs, as well as unwanted environmental impacts resulting from loss of non‐targeted organisms, such as bees, butterflies, and other pollinators. With increased use of insecticides, SWD resistance to organophosphates and pyrethroids is a major threat.^7^, ^8^ Moreover, once eggs have been deposited, insecticidal sprays will no longer protect the fruit, which has been infested with maggots.^2^ It is therefore important to find means of targeting adult female flies to prevent egg‐laying behavior. Here, we evaluate a new environmentally‐friendly species‐specific insecticide that can be deployed as an attractive targeted sugar bait (ATSB) to combat SWD infestations.

Attractive targeted sugar baits utilize the natural sugar feeding behavior of insects such as mosquitoes and flies, many of which require plant sugar for survival and reproduction. ATSBs, which have been developed for the control of vector mosquitoes, capitalize on this natural sugar feeding behavior to attract mosquitoes that feed on a sugar source that has been laced with a poison.^9^ Although ATSBs have not yet been assessed broadly in SWD, trials have demonstrated that ATSBs can significantly reduce sand fly^10^ and mosquito^11^ populations. Sugar baits facilitate targeted delivery of a variety of pesticides,^9^ resulting in an overall reduction in pesticide applications, but resistance is nevertheless still of concern. Furthermore, first‐ generation mosquito ATSBs have centered on the use of non‐specific chemical insecticides that can harm non‐target insects.^12^ To address these concerns, we recently identified a new class of RNA interference (RNAi) pesticides that result in significant mortality in adult mosquitoes of a variety of species when delivered as ATSBs in lab^13^, ^14^ and semi‐field^15^ trials. Use of RNAi insecticides, which are species‐specific, enhances existing ATSB technologies and may represent a new generation of insecticides to combat insecticide resistance.

In the RNAi pathway, a conserved mechanism that helps protect organisms from viral infections, small interfering RNAs (siRNAs) silence (turn off) genes that are complementary in sequence.^16^ This complementarity facilitates the design of custom insecticides.^17^ We have developed multiple RNAi pesticides that target neural genes in various types of mosquitoes.^13^, ^14^ These mosquito‐specific insecticides were designed to target gene sequences conserved in mosquitoes, but not in any other organisms, including humans. Interfering RNA can be expressed in *S. cerevisiae*, a model organism that is genetically tractable, inexpensive to culture at scale, and which can facilitate cost‐effective RNA production during yeast cultivation.^18^ The yeast, which can be heat killed prior to use, is highly attractive to adult mosquitoes, which can consume it through ATSBs delivered in bait station sachets.^15^ Yeast RNAi systems promote high levels of gene silencing in adult mosquitoes, and when used to target genes that are required for mosquito survival, the yeast can generate high levels of mortality upon consumption.^13^, ^14^ Moreover, the yeast formulations developed for mosquito control were shown to maintain activity for several months,^19^ suggesting that a single deployment of yeast ATSBs (rather than weekly treatments) could be sufficient to control SWD throughout a single growing season in the north central United States. Here, we aimed to extend this RNAi yeast ATSB research to develop a new SWD control intervention.

Several of the genes that were successfully targeted by mosquito RNAi pesticides are also present in SWD flies. One example is the *Sh* gene, which functions in the mosquito brain.^20^ *S. cerevisiae* (baker's yeast) was engineered to express interfering RNA corresponding to mosquito *Sh* genes. *Sh* genes encode an evolutionarily conserved subunit of a voltage‐gated potassium channel, a regulator of neural activity in both invertebrate and vertebrate organisms.^21^, ^22^ Consumption of the yeast by mosquitoes silenced *Sh*, resulting in the death of adult females that consumed yeast delivered through a sugar bait. Mortality correlated with defects in the mosquito brain. The insecticidal activities of the yeast were subsequently confirmed in trials conducted on *Aedes albopictus*, *Anopheles gambiae*, and *Culex quinquefasciatus* mosquitoes, yet the yeast was not toxic to non‐target arthropods that lacked the *Sh* target sequence.^15^, ^20^ These studies indicate that yeast RNAi pesticides targeting *Sh* could be further developed as broad‐based mosquito insecticides for utilization in integrated biorational mosquito control programs. These findings indicated that the species‐specificity of ATSBs, a new paradigm for vector control,^9^ could be enhanced through the use of RNAi yeast‐based pesticides.

Although the mosquito yeast RNAi pesticides developed to date do not have a target site in *D. suzukii*, a copy of the *Sh* gene is found in the SWD genome.^23^ We hypothesized that modification of the shRNA sequence such that it matches the SWD version of the *Sh* gene, but not that of other organisms, would allow us to specifically kill SWD. Likewise, recent work^24^ demonstrated that baker's yeast is a potent attractant for SWD in the field, where it performs superiorly to other attractants. Here, we evaluate RNAi yeast targeting the SWD *Sh* gene by incorporating it into an ATSB that was fed to SWD.

## 2 MATERIALS AND METHODS

### 2.1 Insect rearing

An SWD strain prepared from a local Michigan collection was obtained from Juliana Wilson (Michigan State University) and reared in an insectary maintained at 26 °C, ~80% relative humidity, and with a 12 h light/12 h dark cycle with 1 h crepuscular periods. Flies were reared in bottles containing Nutri‐Fly® BF, (Genesee Scientific, El Cajon, CA) media.

### 2.2 Preparation and culturing of Sh.706 yeast

An RNAi yeast strain corresponding to the *D. suzukii* potassium voltage‐gated channel protein‐encoding gene *Sh* (NCBI Gene ID: 108016735) target site CGTTGAGCGGTGAAAACCTATCCAA was generated as previously described^25^ using the *S. cerevisiae CEN.PK* yeast strain (genotype *MATa/α ura3‐52/ura3‐52 trp1‐289/trp1‐289 leu2‐3_112/leu2‐3_112 his3 Δ1/his3 Δ1 MAL2‐8C/MAL2‐8C SUC2/SUC2*).^26^ The *CEN.PK* yeast was transformed with a URA+ pRS426GPD shuttle plasmid^27^ into which the custom *Sh* shRNA expression cassette (synthesized by Invitrogen, Carlsbad, CA, USA) was cloned between the BamH1 and XhoI restriction sites as described.^25^ Yeast transformants were selected through growth on media lacking uracil. The yeast, hereafter referred to as Sh.706, was then shake‐cultured in SCD‐ura media and heat killed upon harvesting as described,^25^ then freeze dried with 0.025% benzoic acid (as a preservative).

### 2.3 Initial RNAi yeast ATSB screening assay

For initial assays to confirm activity of the RNAi yeast insecticide in SWD, 3–4 day old flies were starved for 4–5 h by transferring them into an empty bottle. Control and treatment yeast were prepared. At the end of the starvation period, the bottle of flies was placed on ice for 15–20 min. 100 μL of 10% sucrose solution with 4.5% red food coloring was mixed with 40 mg of yeast using a sterile toothpick. Four ~25 μL drops of the sugar + yeast mixture were placed on a sterile 100 mm × 15 mm Petri dish. Twenty‐five flies were placed on the Petri dish, which was then covered with its lid. Flies were then placed at room temperature (~21 ± 1 °C) for overnight feeding. Feeding was confirmed by the presence of red food dye in the abdomens of the flies. The next morning, flies from each treatment were again placed on ice and then transferred to fresh food vials, in which they were monitored for 6 days. Behavioral phenotypes and survival were assessed. The data were tabulated, analyzed using the Student's *t*‐test, and graphed in Microsoft Excel 365 software. The survival curves were generated using SPSS 25 (IBM, Armonk, NY USA) software by applying the Kaplan–Meier method.

### 2.4 Laboratory ATSB bait station feeding experiments

In an effort to develop a Sh.706 RNAi yeast soda bottle feeder system that could potentially be deployed in the field, a modified MUDUODUO automatic bird feeder cup (Amazon, Seattle Washington) was used to feed yeast to the flies. A small piece of clean dehumidifier filter (Honeywell Home, Charlotte, NC) without the metal layer was inserted along the channel between the feeding area and the reservoir. The channel and reservoir were wrapped with parafilm and covered to minimize leakage. At the feeder end, a platform wick was created using the same filter cut into a 3.3 cm × 7.0 cm rectangle and folded into a circle. Food grade nylon membranes (Amazon, Seattle Washington) of 5 μm (bottom) and 90 μm (top) were layered on top. A paste was prepared by mixing 40–200 mg of either control or treatment yeast with 200 μL of degassed/flat Coca‐Cola and placed between the membrane layers. A soda bottle (12 FL OZ) with 110 mL of Coca‐Cola and 20 mL/L Tegosept anti‐mold agent (Thermo Fisher Scientific, Waltham, Massachusetts) was inverted to act as a continuous supply of soda (Fig. S1). This feeder along with 50 3–4 day old sugar‐starved flies were placed in insect cages. The experiment was set up in the insectary, where it was monitored for 6 days. Mortality and any other phenotypic changes were noted daily. The data were tabulated and graphed in Microsoft Excel 365 software. One‐way ANOVA statistics were performed and survival curves generated using SPSS 25 (IBM, Armonk, NY, USA) software. The Kaplan–Meier method was utilized for the survival curves.

### 2.5 Semi‐field ATSB feeding assays

The feeders and the cages were prepared as described in section 2.4. For each treatment, a feeder along with 50 3–4 day old sugar‐starved flies were placed in the cages. The cages were then placed within a SANSBUG 1‐Person Free‐Standing Pop‐Up Mosquito‐Net (Amazon, Seattle, WA, USA) located within the confines of the Raclin‐Carmichael Hall enclosure, South Bend, Indiana, USA (Fig. 4(F)), where it was monitored for 6 days. Mortality was analyzed daily during the experimental period, which occurred during July 2025. Three replicate trials were performed (150 total flies) in July 2025. The temperature and humidity during the experiment were 23 ± 5 °C and 70 ± 16% relative humidity, respectively. The data were tabulated and graphed in Microsoft Excel 365 software. One‐way ANOVA statistics were performed using SPSS 25 (IMB, Armonk, NY, USA) software.

### 2.6 Confirmation of *Sh* gene silencing

Silencing of the SWD *Sh* gene was examined through qRT‐PCR. Pools of five whole adult flies fed with Sh.706 as described above were collected 16 h after yeast‐ATSB feedings (control or treatment). TRIzol (Invitrogen, Carlsbad, CA) was used to extract total RNA as described in the manufacturer's instructions; the RNA was then treated with DNase using the DNA‐free DNA Removal kit (Invitrogen, Thermo Fisher Scientific, Waltham, MA) according to the manufacturer's instructions. The High Capacity RNA to cDNA Kit (Applied Biosystems, Foster City, CA) was used for production of cDNA, which was amplified in a Bio‐Rad CFX96 Touch Real‐time PCR Detection System (Bio‐Rad, Hercules, CA) using the Power SYBR Green PCR Master Mix (Applied Biosystems, Foster City, CA) and the following primers: Sh1 for: 5’ ATCGAGGAAGACGAGGTGCC 3′ and Sh1 rev: 5’ TCCTCCTCTTCGGCAACGAC 3′. Amplification of *alpha tubulin* was performed with the primers 5′ AGGATGCGGCGAATAACT 3′ (forward) and 5′ CGGTGGATAGTCGCTCAA 3′ (reverse)^28^ and used for data standardization. PCR reactions were performed in six replicate wells in each of three separate biological replicate trials. Results from these assays were quantified through standardization of reactions to levels of *alpha tubulin* using the ΔΔCt method as described.^29^ Data were statistically analyzed using Microsoft Excel 365 software with Student's t‐test.

### 2.7 Immunohistochemistry studies

Immunohistochemical staining of adult fly brains was performed after Sh.706 or control treatments using the following reagents as described^30^, ^31^: mAb nc82 anti‐Bruchpilot^32^ (DSHB, Iowa Hybridoma Product nc82 deposited by E. Buchner) which labels active synapses, anti‐HRP (Jackson ImmunoResearch Labs, West Grove, PA) which labels neurons, and TO‐PRO‐3 iodide (Molecular Probes, Eugene, OR) which marks nuclei. The staining assays were performed in triplicate, and stained tissues were mounted and imaged using a Zeiss 710 confocal microscope. Confocal images were analyzed with FIJI ImageJ^33^ and Adobe Photoshop 2025 software; mean gray values (average signal intensity over the selected area) were calculated as previously described^34^ and compared using one‐way ANOVA and Bonferroni post‐hoc test in SPSS 25 (IBM, Armonk, NY, USA) software. The data were graphed in Microsoft Excel 365 software.

### 2.8 Dose–response curves

Dose–response assays were conducted as described in mosquitoes^35^ through the generation of different concentrations of ATSB yeast (0–400 μg/μL) using varying amounts of control and treatment RNAi yeast. The experiments were carried out with 25 individuals/treatment in a petri dish as described above. The final data were tabulated, and probit analyses were performed with SPSS 25 (IBM, Armonk, NY, USA) software. The data were graphed in Microsoft Excel 365 software.

### 2.9 Non‐target assays

The impacts of Sh.706 yeast feedings on non‐target dipterans were assessed in *Aedes aegypti, Anopheles stephensi*, and *Culex quinquefasciatus* mosquitoes. Mosquitoes were reared, and yeast sugar feeding assays were performed as described^13^ using a setup for mosquitoes which is comparable to that used for SWD cage feedings.

## 3 RESULTS

### 3.1 Adult fly consumption of yeast expressing shRNA targeting SWD *Sh* results in insect mortality

**Figure 1:**
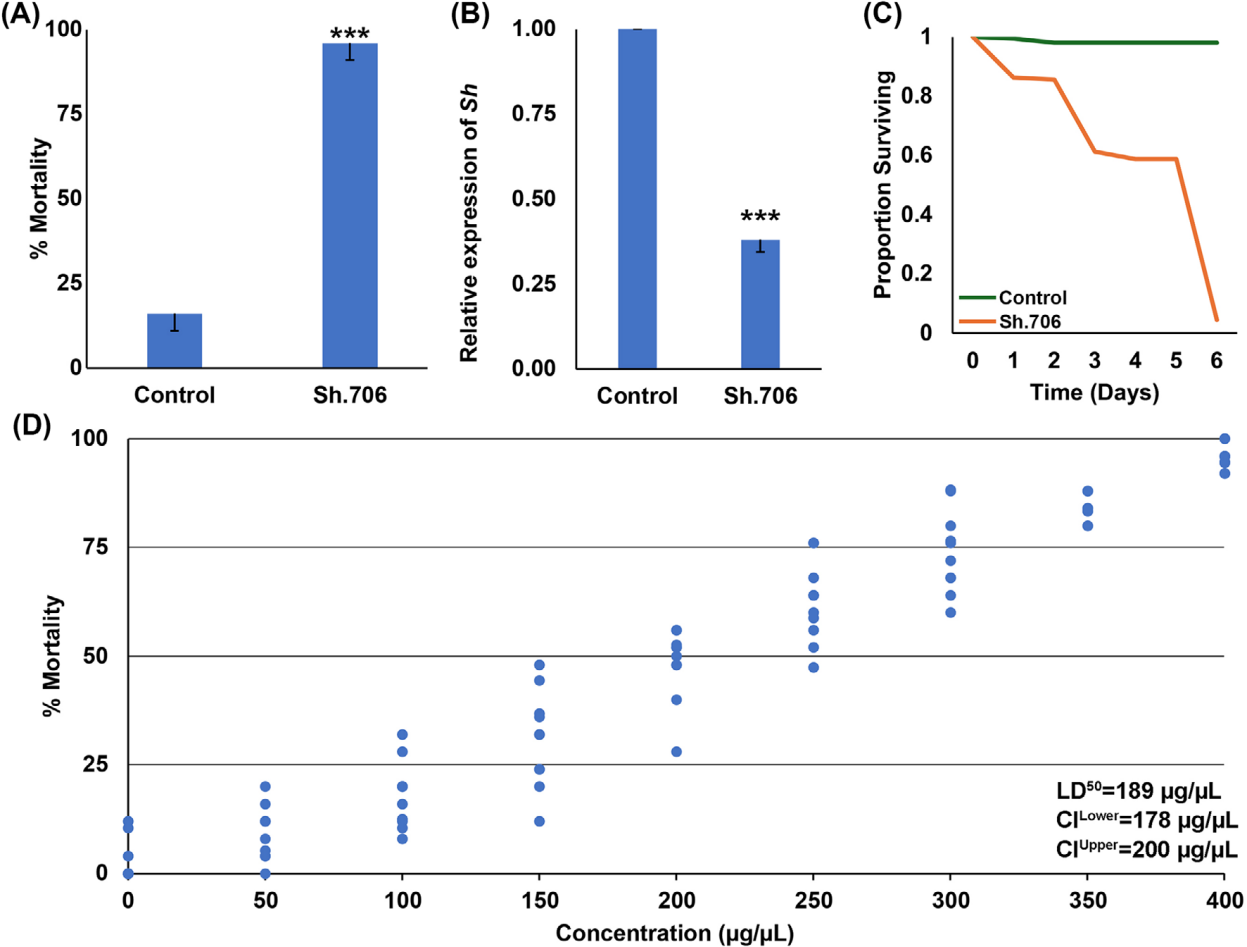
Insecticidal activity of Sh.706 yeast. (A) Laboratory experiments showed that yeast expressing Sh.706 shRNA caused significant mortality in SWD flies compared to those fed with yeast expressing control shRNA with no target specificity (***P < 0.001, Student's *t*‐test; error bars represent the standard error of the mean, SEM). (B) qRT‐PCR confirmed that the SWD *Shaker* gene was silenced in flies that consumed Sh.706 yeast compared to those fed with control yeast (***P < 0.001, Student's *t*‐test; error bars represent SEM); a survival curve is shown in (C). (D) Dose‐dependent mortality was observed in *D. suzukii*, with an LD_50_ of 189 μg/μL. Data were compiled from 10 replicate trials per condition, each containing 25 adults.

*CEN.PK* strain RNAi yeast expressing shRNA corresponding to the SWD *Sh* gene from the pRS426 GPD plasmid was prepared and assessed for insecticidal activity. Although the Sh.706 yeast corresponds to a target sequence present in the *D. suzukii* and *D. melanogaster Sh* genes, NCBI blast searches did not identify other organisms with an identical 25 bp target site with the exception of *D. melanogaster*, which lacks a systemic RNAi response. Following culturing, heat‐inactivation, and drying of the yeast, it was mixed with sucrose and offered to SWD in initial screening assays conducted in petri plates to confirm insecticidal activity of the yeast. Although SWD fed with sucrose containing a control yeast strain^35^ that expresses shRNA with no known target in SWD survived, significant mortality (96 ± 9%, *P* < 0.001 vs. control, Fig. 1(A)) was observed within 6 days (Fig. 1(C)) after SWD consumed the Sh.706‐sucrose bait. Dose–response assays for Sh.706 in SWD generated the curve shown in Fig. 1(D), which revealed an LD_50_ of 189 μg yeast/μL of 10% sucrose.

### 3.2 Silencing of *Sh* expression results in neural and behavioral phenotypes in adult flies

**Figure 2:**
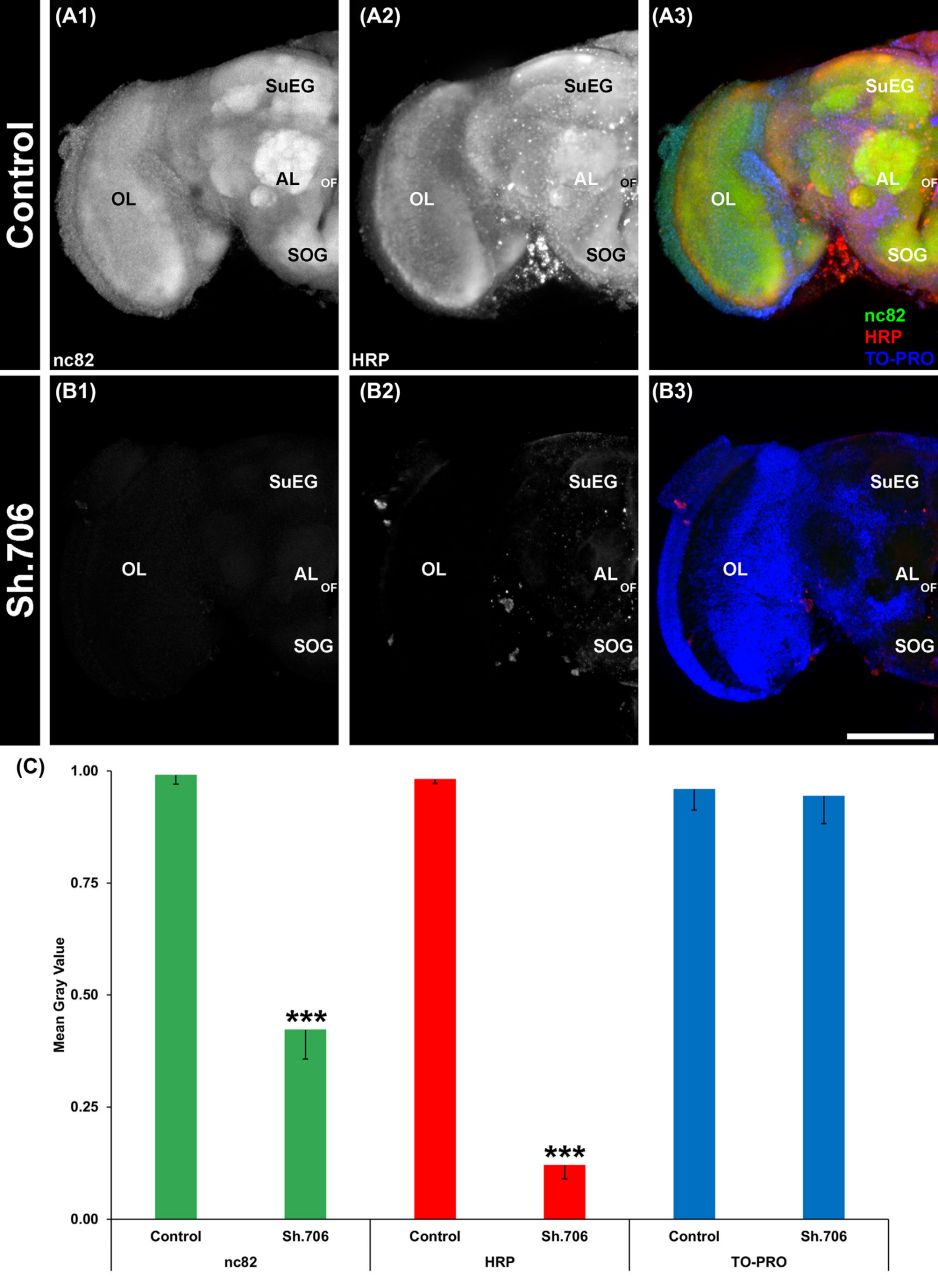
Neural defects in *D. suzukii* adults fed with Sh.706 yeast. Adult brains were labeled with three antibodies: mAbnc82 (active synapse marker; white in (A1, B1); green in (A3)), anti‐HRP antibody (neural marker; white in (A1, B2); red in (A3)), and TO‐PRO (nuclear marker; blue in (A3, B3)). The levels of nc82, a protein involved in neural development, were reduced by ~50% in the synaptic neuropil of adults fed with Sh.706 yeast compared to control yeast‐treated adults (A1 vs B1, C‐green bars). The general neural marker HRP also showed reduction in expression (~80% reduction) compared to control‐fed individuals (A2 vs B2, C‐red bars). Data are presented as average mean gray values, with error bars denoting the standard error of the mean. ***Indicates a statistically significant difference from the control group (*P* < 0.001). Representative larval brains are oriented dorsal upward. AL, larval antennal lobe; OL, optic lobe; SOG, sub‐esophageal ganglion; SuEG, supraesophageal ganglion. A combined total of 57 control and 63 Sh.706‐treated individual brains from three biological replicate experiments were analyzed. Scale Bar = 100 μm.

It was hypothesized that neural defects would be observed in SWD flies that had consumed the RNAi yeast ATSB. 60% silencing of *Sh* expression, which was verified through qRTPCR (Fig. 1(B)), correlated with neural (Fig. 2) and behavioral defects. *Sh* silencing correlated with a significant 58% reduction in levels of nc82, which labels Bruchpilot, a marker of active synapses^32^ in the adult brain (*P* < 0.001, Fig. 2 (A1 vs B1) and (C), green). In addition, the anti‐HRP labeled brains showed a significant ~80% reduction in expression when compared with control brains (*P* < 0.001, Fig. 2 (A2 vs B2) and (C), red). However, no significant differences in TO‐PRO nuclear staining were observed in treated and control fly brains (*P* > 0.05, Fig. 2 (A3 vs B3) and (C), blue). These flies, which displayed significant neural defects following Sh.706 yeast treatment, were also assessed for behavioral defects. Shaking was observed in the legs of treated flies, which had locomotor defects and were unable to fly.

### 3.3 Sh.706 yeast consumption does not impact non‐target dipterans

**Figure 3:**
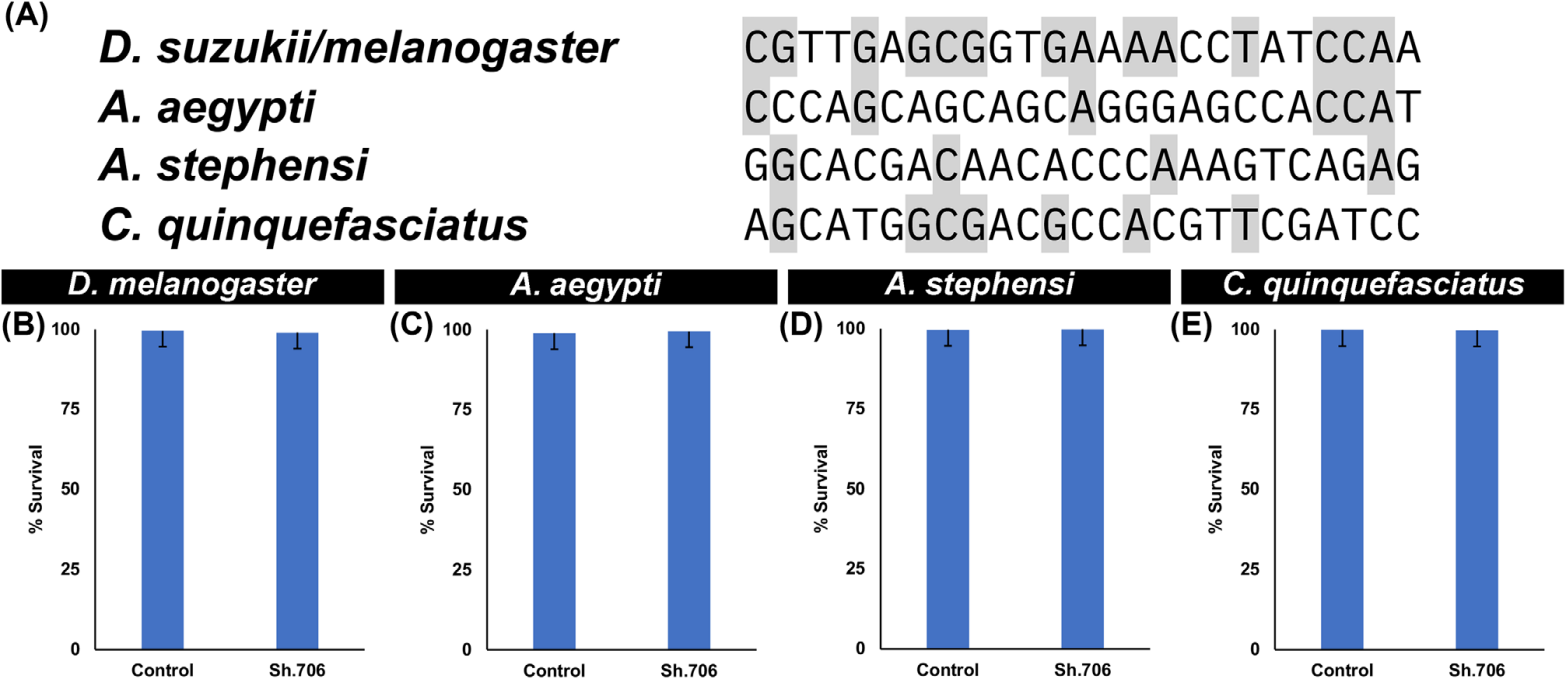
Survival of non‐target arthropods consuming Sh.706 yeast. (A) The *D. suzukii/melanogaster Sh.706* target site sequence is not conserved in mosquitoes. Alignments between the *D. suzukii*/*melanogaster Sh.706* target site and corresponding sites in the mosquito *Sh* genes are shown, with conserved bases highlighted in grey. Consumption of Sh.706 yeast by *D. melanogaster* (B), *A. aegypti* (C), *A. stephensi* (D), and *C. quinquefasciatus* (E) adults did not affect non‐target insect survival. A total of 150 adults per treatment were assessed in two biological replicate experiments for all species. Graphs display mean survival percentages with standard deviation as error bars. Student's *t*‐test was used to compare means of control‐fed vs treatment‐fed individuals.

Despite the significant neural and behavioral defects observed in SWD, it was hypothesized that consumption of Sh.706 yeast would not impact survival of non‐target dipterans. Although the Sh.706 target site is conserved between *D. melanogaster* and *D. suzukii* (Fig. 3(A)), *D. melanogaster*, which lack systemic RNAi,^36^ did not die (Fig. 3(B)). The Sh.706 target site is not well conserved in the mosquito *Sh* genes (Fig. 3(A)). Alignments of the SWD Sh.706 target site in the *D. suzukii Sh* gene and corresponding regions in the mosquito *Sh* genes (Fig. 3(A)) demonstrate the lack of a conserved Sh.706 site in the *A. aegypti* (6/25 identical bases, 24% nucleotide identity), *A. stephensi* 4/25 identical bases, 16% nucleotide identity, and *C. quinquefasciatus* (7/25 identical bases, 28% nucleotide identity) *Sh* genes. No significant death (*P* > 0.05) was detected in the survival of adult *A. aegypti, A. stephensi*, and *C. quinquefasciatus* adult mosquitoes that consumed Sh.709 yeast or control yeast (Fig. 3(C)–(E)), all of which survived.

### 3.4 Development of an RNAi yeast ATSB bait station

**Figure 4:**
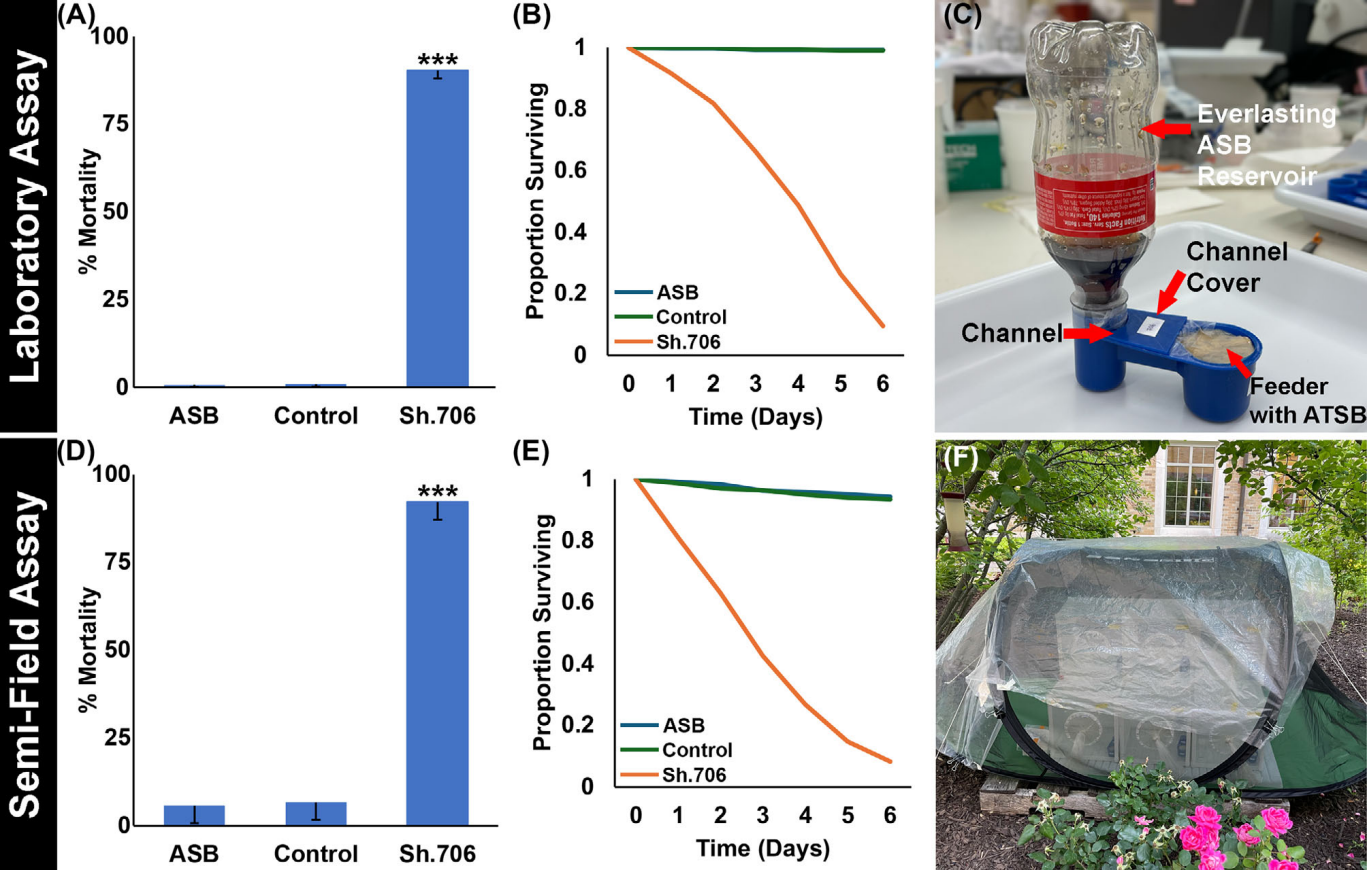
Sh.706 ATSB delivered in a YES feeder induced significant mortality. Sh.706 yeast fed to *D. suzukii* adults as an ATSB prepared with soda and delivered in the YES feeder led to significant mortality when compared with control yeast‐fed individuals or soda alone (A; a survival curve is shown in B). The YES feeder consists of automatic bird feeder cups modified to deliver yeast ATSB prepared with soda (C). A combined total of 506, 537 and 514 individuals were tested from four biological replicate experiments for ASB, control, and Sh.706 treatments, respectively (A, B). Comparable results (D; survival curve in E) were observed in semi‐field trials performed in an outdoor enclosure (F) in South Bend, IN during July 2025), again with significant mortality rates observed in Sh.706‐treated flies. Three replicate semi‐field trials, each with 50 flies/treatment (total of 150 flies/treatment) were performed.

Soda has previously been shown to function as a sugar bait that can attract mosquitoes.^37^ In an effort to create a yeast delivery system that could be easily constructed and evaluated in the field, a Sh.706 yeast‐soda feeder system, deemed the yeast everlasting soda (YES) feeder, was prepared (Fig. S1) and assessed in the insectary under simulated field conditions (Fig. 4(A),(B)). All flies were observed drinking from the feeders, which were prepared either with soda alone or with soda and Sh.706 or control RNAi yeast. Although flies that drank from the Sh.706 feeder died within 6 days, flies that consumed the control yeast or soda alone survived (*P* < 0.001 vs. control yeast or soda alone, Fig. 4(A),(B)). The YES feeders were also assessed in semi‐field trials completed in screened tents placed in outdoor enclosures (Fig. 4(F)). Comparable results were observed in these semi‐field trials (Fig. 4(F)), in which control yeast‐treated flies or flies that drank soda alone survived, but flies that consumed the Sh.706 yeast ATSB died (*P* < 0.001 vs control yeast or soda alone, Fig. 4(D)) within 6 days (Fig. 4(E)).

## 4 DISCUSSION

### 4.1 Demonstration that Sh.706 is an eco‐friendly RNAi yeast insecticide

Jarausch *et al*.^38^ discussed the great potential, but critical challenges for the development of RNAi control measures for SWD. Among the challenges, they note the need for an effective delivery system for RNAi pesticides. They also discuss the necessity of identifying the proper target genes. In this investigation, which successfully addressed both these challenges, Sh.706, an RNAi yeast pesticide that silences *Sh*, a gene required for SWD survival, was produced at low cost in baker's yeast that was heat‐inactivated, dried with a preservative, and fed to SWD using attractive sugar baits (Fig. 1). Consumption of the Sh.706 yeast ATSB resulted in insect mortality (Fig. 1), which correlated with silencing of *Sh* (Fig. 1(B)) and defective neural activity in the fly brain, as demonstrated through loss of nc82 and HRP signal that likely results from loss of both neural activity and neural density (Fig. 2). These significant neural defects, accompanied by shaking phenotypes, correlated with the significant mortality observed in Sh.706‐treated flies (Fig. 1). The Sh.706 yeast was then mixed with soda and successfully delivered to SWD in an inexpensive, easy to assemble and use soda bottle feeder (Fig. 4, Fig. S1). It is anticipated that the yeast ATSB feeder system (Fig. 4(C)) that performed well in both indoor (Fig. 4(A),(B)) and outdoor semi‐field trials (Fig. 4(D)–(F)), could be rapidly deployed and seamlessly integrated with existing SWD strategies, while enhancing these strategies through inclusion of a novel class of SWD‐specific pesticides that does not impact non‐target dipteran insect survival (Fig. 3).

RNAi resulting from dsRNA synthesis and delivery in a microbe was originally discovered in the nematode *Caenorhabditis elegans*,^39^ which possesses RNA‐dependent RNA polymerases (RdRps) that trigger synthesis of secondary RNAs that amplify the RNAi signal and sustain a systemic RNAi response beyond the gut. *Drosophila* lack canonical RdRPs,^36^ and the direct ingestion of naked dsRNA in *D. melanogaster* ^40^ or *D. suzukii* ^41^ does not yield systemic RNAi responses unless delivery vehicles, such as liposomes, are utilized. In addition to a lack of RdRps in *D. suzukii*, it is speculated that two dsRNAses expressed at high levels within the gut lead to destruction of naked dsRNAs upon consumption, thereby blocking a systemic RNAi response in SWD.^42^ Murphy *et al*.^43^ generated insecticidal yeast strains expressing long dsRNAs targeting genes expressed within the *D. suzukii* gut, where it was hypothesized that RNAi activity would be most prominent; feedings with the live yeasts from these strains resulted in SWD death. Here we show that delivery of shRNA by heat‐inactivated yeast results in *D. suzukii* death and defects that were observed beyond the gut, within the central nervous system (Fig. 2). The neural defects observed in this investigation, and in a second investigation in which yeast RNAi‐mediated silencing of the SWD *Rbfox1* gene elicited neural defects,^44^ suggest that this inactivated yeast shRNA delivery system results in silencing beyond the *D. suzukii* gut. The yeast RNAi system appears to be a robust method for characterizing gene function within the *D. suzukii* central nervous system and could perhaps be useful for studies in other tissues.

Although *D. melanogaster* and *D. suzukii* share the same Sh.706A target sequence (Fig. 3(A)), *D. melanogaster* did not die when fed with the same yeast that killed *D. suzukii* (Fig. 4). This result suggests that in *D. suzukii*, yeast‐mediated delivery of shRNA protects it from being degraded by gut dsRNAses and may help improve interfering RNA uptake (presumably *via* endocytosis), intracellular transport, and/or endosomal escape of dsRNA, key processes that determine RNAi efficiency in various insects.^45^ No differences in the presence/absence of genes encoding components of these RNAi processes have been noted in *D. melanogaster* or *D. suzukii*, but perhaps differences in the timing, levels, or tissue specificity of such genes or the activities of their protein products are present. These topics could be explored in future research studies.

### 4.2 Semi‐field trials provide impetus for larger field trials

Yeast RNAi ATSBs were originally developed as a potential new tool for mosquito control, for which several yeast strains targeting various genes required for adult survival have been targeted.^13^, ^14^ The results of this investigation suggest that RNAi yeast ATSBs could potentially be explored as a tool for SWD control. To this end, a soda bottle system was developed for delivery of an RNAi yeast soda ATSB. The system was effective in both indoor simulated field (Fig. 4(A)–(C)) and outdoor semi‐field (Fig. 4(D)–(F)) trials. The high mortality rates observed in outdoor semi‐field trials (Fig. 4(D)–(F)), suggesting that the yeast maintains sufficient insecticidal activity in an outdoor setting. These semi‐field results provide impetus for the pursuit of larger‐scale field trials to further assess the efficacy of the RNAi yeast ATSB feeder system. The YES feeder system (Fig. 4(C)) is relatively inexpensive and easy to construct, making it suitable for larger trials and potentially as a commercial product. The feeder systems performed well in trials in which the ATSBs were the only sources of sugar bait (Fig. 4), but this system should also be assessed in the presence of natural competing sugar sources. Moreover, given the need to target adult SWD flies prior to egg deposition in fruits,^38^ field trials should also be designed to assess the best time and location(s) for deployment of the RNAi ATSB bait stations.

The success of the outdoor SWD semi‐field trials (Fig. 4(D)–(F)) suggests that the ATSB, which contains a preservative, maintains sufficient insecticidal activity following exposure of the ATSB to sun, summer heat, and outdoor elements. Similarly, RNAi yeast ATSBs targeting mosquitoes maintained insecticidal activity in outdoor semi‐field trials conducted in both Trinidad and Thailand.^15^ Moreover, RNAi yeast tablets targeting mosquitoes maintained insecticidal activity against *A. aegypti* larvae for 6 months when deployed in large water storage containers located in an outdoor rooftop laboratory in Trinidad.^19^ These studies suggest that the RNAi yeast insecticides maintain RNAi activity over time and when exposed to outdoor elements, providing further impetus for the pursuit of larger field trials in which the efficacy of the YES feeder system can be further assessed.

As RNAi ATSBs are further evaluated in the field, it will also be critical to conduct engagement activities with relevant stakeholders in areas where the trials are conducted. Such activities, which were performed in conjunction with mosquito yeast RNAi field trials,^46^ will promote trust and cooperation between farmers and scientists, build a community united to combat SWD, allow stakeholders to provide input into product development, and increase access to the new technology by identifying farms to participate in future scaled product testing.^47^ Through early and ongoing stakeholder engagement, a highly effective, stakeholder‐accepted new SWD control product could be produced. It is anticipated that environmentally‐friendly yeast ATSB pesticides, which are projected to be cost‐competitive and readily produced at scale,^18^, ^48^ will help promote sustainable SWD control.

### 4.3 RNAi yeast design: implications for scale‐up and regulatory considerations

The yeast evaluated in this proof‐of‐principle study were transformed with a plasmid that enabled expression of the insecticidal Sh.426 shRNA. Even though the yeast is heat‐killed prior to feeding SWD, for field trials, it would be better to move toward a yeast strain with shRNA expression cassettes that have been integrated into a yeast genome. This would eliminate use of a plasmid with an antibiotic resistance marker,^27^ which is likely more acceptable to regulatory bodies. Moreover, instead of the *CEN.PK* ^26^ laboratory yeast strain used in this investigation, it would be better to use a robust yeast strain that is suitable for larger‐scale fermentations. One method for generating such strains was recently discussed by Brizzee *et al*.,^48^ who used Cas‐CLOVER and Super PiggyBac engineering in *S. cerevisiae* to integrate multiple copies of an insecticidal shRNA expression cassette into the yeast genome. A 30‐fold increase in shRNA production was observed in the new strain, which reduces the amount of yeast that must be consumed to result in mosquito death and is expected to further reduce the cost of the intervention. Moreover, this Cas‐CLOVER strain performed well in pilot scaled fermentations, in which specialized, expensive media were not required, suggesting that scaling yeast production will be both straightforward and inexpensive.

It should be noted that although others have discussed a symbiotic yeast RNAi system that requires the use of live yeast,^43^ the yeast pesticides used in the present investigation were heat‐inactivated. This is expected to significantly alleviate potential regulatory concerns associated with the release of live RNAi yeast and permits classification of the yeast insecticide as a dead microbial. Moreover, this investigation is the first to combine the specificity of the Sh.706 RNAi yeast pesticide (Fig. 3) with the attractiveness of a sugar bait for SWD flies, resulting in an SWD‐specific ATSB. This new class of RNAi pesticides could help alleviate insecticide resistance while posing little if any threat to non‐target organisms. The use of bait stations also allows the insecticides to be delivered in a targeted manner, which greatly reduces the indiscriminate use of pesticides.

Field trials will enable identification of the ideal deployment locations for bait stations (Fig. 4) such that they cannot be disrupted. In the large‐scale Westham mosquito ATSB bait station trials in Africa,^12^ the bait stations were hung on the upper walls of participants' dwellings. Heads of households reported that other insects and a rodent attempted to feed at the stations.^49^ Although the Sh.706 ATSB was found to be non‐toxic when fed to several non‐target insects (Fig. 3), having to replace the stations frequently as a result of tampering from rodents or other scavengers would not be desirable. Moreover, while the Sh.706 yeast was designed to be safe for non‐target organisms (Fig. 3), further toxicity assessments will likely be needed for the pursuit of regulatory authority approvals and to ensure that the RNAi yeast ATSB system achieves the goal of eradicating SWD in a sustainable and environmentally‐conscious manner.

The soda formulations with added mold‐inhibitor worked well in both lab trials and outdoor semi‐field trials (Fig. 4), but it will be critical to further assess their efficacy in the field. In the event that these lures do not perform well, other lures could also be assessed. For example, a quinary blend that is both efficient and selective for SWD was identified^50^ and could be evaluated in conjunction with RNAi yeast. Previous baited traps have also made use of pheromones to lure SWD.^51^ The inclusion of such pheromones could further increase the attractiveness of the ATSBs to SWD. This will be critical in the field, where competitive natural sugar sources are also available. Moreover, in the future, SWD repellents could potentially be combined with the ATSB technology to develop push‐pull strategies.^52^ Finally, the development of a spray formulation, which could be applied to competing non‐crop sugar sources, could also prove useful. *Bacillus thuringiensis israelensis* (Bti) bacterial larvicide spray applications have become important aspects of integrated vector control programs for mosquito control,^53^ suggesting that development of spray RNAi yeast formulations could be successfully pursued.

## 5 CONCLUSIONS

The findings of this study demonstrate that a species‐specific RNAi yeast insecticide can serve as a highly toxic component of an ATSB that effectively kills *D. suzukii*, an invasive fruit and berry pest. The yeast ATSB, which silences the *Sh* gene resulting in severe neural and behavioral phenotypes, can be prepared using soda delivered in an inexpensive easily constructed soda bottle feeder which effectively killed SWD under simulated field and semi‐field conditions. Although further evaluation of the RNAi yeast ATSB feeders in the field is needed, RNAi yeasts targeting SWD may represent a new class of effective, yet biorational insecticides that can be used in integrated pest management programs for control of this destructive insect pest.

## CONFLICTS OF INTEREST

MDS and KM have a pending U.S. patent application related to this work, but this application did not impact their analysis of the data described herein. All other authors have no conflicts of interest. The *Drosophila suzukii* photographs can be used and were posted by Shane McEvey (CC BY 4.0) McEvey S, High Resolution Diagnostic Images of *Drosophila suzukii* (Diptera: Drosophilidae), figshare (2017), available at: https://figshare.com/articles/figure/High_resolution_diagnostic_images_of_Drosophila_subpulchrella_Diptera_Drosophilidae_/5363431?file=9217795.

## Supporting information


**Figure S1.** Steps demonstrating construction of the YES feeder prepared for delivery yeast‐soda ATSBs to *D. suzukii*.

## Data Availability

All data is available within the manuscript and supplementary material.
