# Supplementary figures and images for: Development of an eco‐friendly RNAi yeast attractive targeted sugar bait that silences the shaker gene in spotted‐wing drosophila, Drosophila suzukii
Source: Pest Manag Sci. 2025 Sep 18;82(1):732–41. doi: 10.1002/ps.70228 (PMC12713712; doi:10.1002/ps.70228)

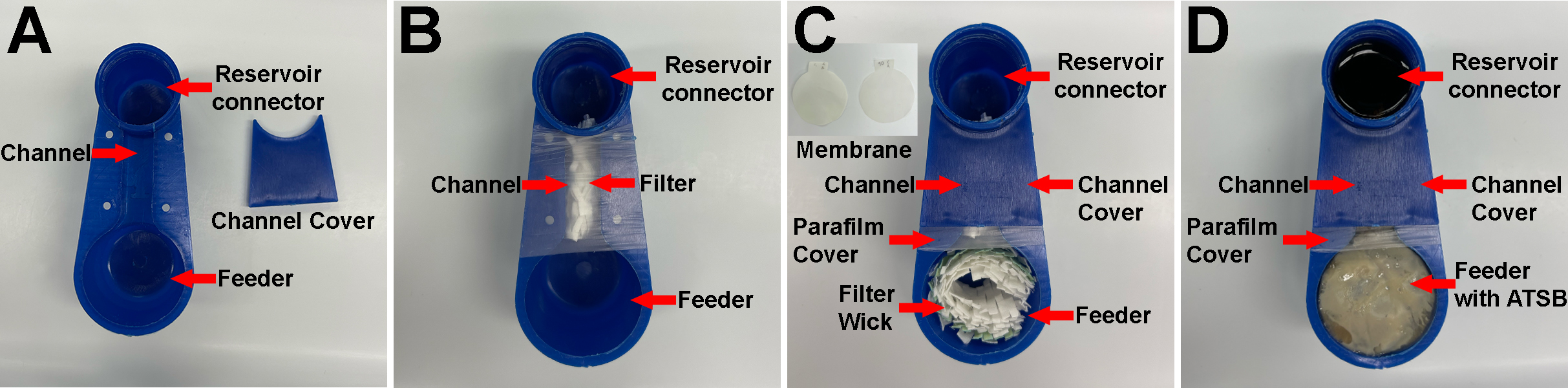

Supplement: Supplementary file 1 — Figure S1. Steps demonstrating construction of the YES feeder prepared for delivery yeast‐soda ATSBs to D. suzukii. [file PS-82-732-s001.jpg]
